# A non-enhanced CT-based deep learning diagnostic system for COVID-19 infection at high risk among lung cancer patients

**DOI:** 10.3389/fmed.2024.1444708

**Published:** 2024-08-12

**Authors:** Tianming Du, Yihao Sun, Xinghao Wang, Tao Jiang, Ning Xu, Zeyd Boukhers, Marcin Grzegorzek, Hongzan Sun, Chen Li

**Affiliations:** ^1^College of Medicine and Biological information Engineering, Northeastern University, Shenyang, China; ^2^Department of Radiology, Beijing Friendship Hospital, Capital Medical University, Beijing, China; ^3^Institute of Medical Informatics, University of Lübeck, Lübeck, Germany; ^4^Department of Radiology, Shengjing Hospital of China Medical University, Shenyang, China; ^5^Institute of Intelligent Medicine, Chengdu University of Traditional Chinese Medicine, Chengdu, China; ^6^School of Arts and Design, Liaoning Petrochemical University, Fushun, Liaoning, China; ^7^Fraunhofer Institute for Applied Information Technology FIT, Sankt Augustin, Germany; ^8^German Research Center for Artificial Intelligence, Lübeck, Germany

**Keywords:** Data mining, COVID-19, CT, deep learning, KL-6

## Abstract

**Background:**

Pneumonia and lung cancer have a mutually reinforcing relationship. Lung cancer patients are prone to contracting COVID-19, with poorer prognoses. Additionally, COVID-19 infection can impact anticancer treatments for lung cancer patients. Developing an early diagnostic system for COVID-19 pneumonia can help improve the prognosis of lung cancer patients with COVID-19 infection.

**Method:**

This study proposes a neural network for COVID-19 diagnosis based on non-enhanced CT scans, consisting of two 3D convolutional neural networks (CNN) connected in series to form two diagnostic modules. The first diagnostic module classifies COVID-19 pneumonia patients from other pneumonia patients, while the second diagnostic module distinguishes severe COVID-19 patients from ordinary COVID-19 patients. We also analyzed the correlation between the deep learning features of the two diagnostic modules and various laboratory parameters, including KL-6.

**Result:**

The first diagnostic module achieved an accuracy of 0.9669 on the training set and 0.8884 on the test set, while the second diagnostic module achieved an accuracy of 0.9722 on the training set and 0.9184 on the test set. Strong correlation was observed between the deep learning parameters of the second diagnostic module and KL-6.

**Conclusion:**

Our neural network can differentiate between COVID-19 pneumonia and other pneumonias on CT images, while also distinguishing between ordinary COVID-19 patients and those with white lung. Patients with white lung in COVID-19 have greater alveolar damage compared to ordinary COVID-19 patients, and our deep learning features can serve as an imaging biomarker.

## 1 Introduction

Lung cancer has the highest incidence and mortality rates among malignant tumors. According to the 2018 GLOBOCAN cancer database report, lung cancer accounts for 11.6% of all cancer cases and is the leading cause of cancer deaths worldwide for both men and women, making up 18.4% of all cancer-related deaths (1). Lung cancer typically leads to death due to various complications such as asphyxiation, hypovolemic shock, and multiple organ failure (2). Pneumonia is not only one of the leading causes of death from lung cancer, but infections can also increase the risk of developing lung cancer (3–5). In chronic pneumonia, the infiltration of inflammatory cells and the accumulation of pro-inflammatory factors, including cytokines, prostaglandins, and chemokines, can stimulate various physiological processes, including cell proliferation, angiogenesis, and metastasis (6). Therefore, although there is no direct evidence proving that pneumonia is the fundamental cause of lung cancer, lung infections may contribute to the formation of an inflammatory environment conducive to the occurrence and development of lung cancer.

Originating in 2019, Corona Virus Disease 2019 (COVID-19), which can quickly cause severe acute respiratory syndrome and fatal pneumonia (7). The most common symptoms of COVID-19 infection are fever, dry cough, difficulty breathing, headache, and pneumonia. The progression of the disease may lead to gradually worsening respiratory failure and can even be fatal (8). Since COVID-19 has the potential to trigger a cytokine storm, patients with severe pneumonia may be at risk of developing multiple organ failure (9). This ultimately results in congestion and edema of the alveolar septa, focal hemorrhage and necrosis of lung tissue, alveolar exudation, and the formation of pulmonary interstitial fibrosis (10). Among these, the symptoms of pulmonary fibrosis are particularly prominent (11). Studies have shown that patients without lung cancer can develop related chronic inflammation after contracting COVID-19, which then stimulates and damages alveolar epithelial tissue, resulting in pulmonary fibrosis and potentially leading to lung cancer (12). Cancer patients, especially those with lung tumors, are more susceptible to COVID-19 infection (13). Studies have indicated that this is a result of the interaction between angiotensin-converting enzyme 2 (ACE2) in the body and COVID-19 (14, 15). This mechanism exacerbates the symptoms in lung cancer patients with concurrent COVID-19 infection and increases the transmission risk of COVID-19 (9). Therefore, lung cancer patients have a higher risk of developing severe illness and death after contracting COVID-19 (13). In addition, patients with combined lung cancer may also face an increased risk of death due to systemic immunosuppression caused by the cancer itself and anticancer treatments (16).

Currently, COVID-19 patients can receive standardized treatment protocols, greatly improving patient prognosis (17). However, as the COVID-19 pandemic subsides, infected individuals often lack clearly traceable infection paths, making it difficult to diagnose epidemiologically as during the peak of the COVID-19 pandemic. Additionally, Lung cancer patients are inherently prone to bacterial infections (18, 19). This results in lung cancer patients often finding it challenging to undergo accurate diagnosis through simple procedures in the early stages of the disease, ultimately making them more prone to progressing to severe COVID-19 pneumonia (20). Due to the traditional lack of early specificity in COVID-19’s radiological imaging, early diagnosis of COVID-19 pneumonia presents a challenge (21). Moreover, the results of most blood tests are usually nonspecific, with significant variability among different ethnic groups and stages of disease (22). Therefore, the diagnosis of COVID-19 is typically based on nucleic acid tests, immunoassays, radiology, and biosensor methods (23). Studies have shown that chest computed tomography (CT) can capture the typical radiological features of COVID-19 patients (24). Artificial intelligence methods can significantly improve the accuracy of chest CT diagnoses (25–27).

This study developed an automatic diagnostic system based on patients’ CT images using convolutional neural networks (CNN). The deep learning features generated by this model can automatically differentiate between COVID-19 pneumonia and non-COVID-19 pneumonia without the need for manual annotation by clinical doctors. Furthermore, the system can predict whether COVID-19 pneumonia patients will develop into severe pneumonia. Finally, we established medical interpretations of the deep learning features using Krebs Von den Lungen-6(KL-6), a serum biomarker highly associated with lung tissue damage, and explored the predictive ability of these deep learning features for the course of COVID-19 patients. This aids in improving the prognosis of lung cancer patients infected with COVID-19.

## 2 Materials and methods

### 2.1 Inclusion criteria

**FIGURE 1:**
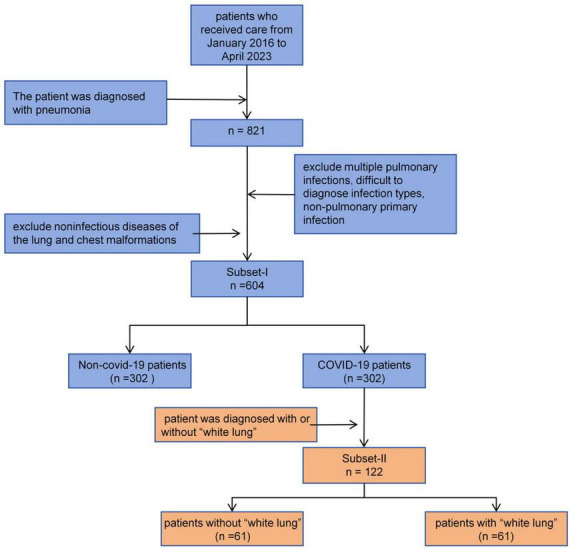
Inclusion and exclusion criteria for patients in this study.

In this study, we prepared two retrospective databases, namely Subset-I and Subset-II:

Subset-I was used for the differential diagnosis of early COVID-19 pneumonia and other types of pneumonia. We searched the imaging database of China Medical University, focusing on patients who visited Shengjing Hospital of China Medical University from January 2016 to May 2023. We included patients diagnosed with bacterial pneumonia, mycoplasma pneumonia, allergic pneumonia, obstructive pneumonia, COVID-19 pneumonia, and other viral pneumonias. For other viral pneumonias, we selected patients from before 2022 to ensure they did not have concurrent COVID-19 infection. All patients were diagnosed through serology rather than symptoms. All included patients had at least one CT scan within a week of symptom onset. We excluded patients who could not be diagnosed with a single pathogen infection and those who were already in the mid or late stages of the disease at the time of their visit, as early warning significance of the CT scans would be lost. Subset-II was used for classifying COVID-19 patients. Each patient had at least two CT scans to determine whether they had progressed to severe pneumonia, also known as “white lung”. The inclusion and exclusion criteria for patients are shown in Figure 1.

### 2.2 CT imaging protocol

A 320-channel scanner (Aquilion ONE 640; Canon Medical Systems) and a 256-channel scanner (Brilliance 128; Philips Medical Systems) were used. The imaging parameters were as follows: tube current 80–230 mA, tube voltage 120 kV, slice thickness 1–3 mm, FOV 500 mm, detector spacing 0.75–1.172 mm.

### 2.3 White lung diagnosis

White lung is defined as an increase in lung lesion area greater than 50% between two CT scans (usually within 24–48 h). Additionally, a high-density area covering more than 70% of the lung on CT images is also defined as white lung. All white lung diagnoses were made by two radiologists with over 6 years of clinical experience and reviewed by a senior radiologist with 11 years of experience. If there was any disagreement in the diagnosis, it was resolved through consultation between the radiologists.

**FIGURE 2:**
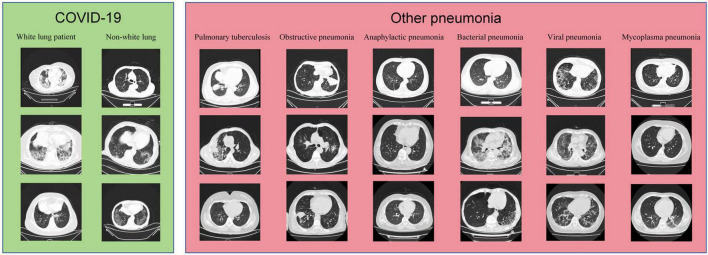
Samples of data used in this study.

The samples from the Subset-I and Subset-II in this study are shown in Figure 2.

### 2.4 Laboratory parameters

We preserved serum samples from some patients and selected the earliest sample from each patient for Laboratory testing. The blood cell count was performed using the electrical impedance method, white blood cells were classified using the VCS method, D-dimer (DD) was measured by the latex agglutination method, and procalcitonin (PCT), C-reactive protein (CRP), and interleukin-6 (IL-6) were measured using immunofluorescence.

The KL-6 test kit was produced by Jiangsu Baweis Biotech Co., Ltd., batch number 23010301, and the testing method was latex immunoturbidimetric assay. Before sample testing, instrument parameters were set, as shown in Table 1. Instrument calibration and reagent calibration were passed, and indoor quality control was under control.

**TABLE 1:** KL-6 Testing Parameters.

Parameter Name	Setting value
Sub/Main Wavelength	−/570nm
Methodology	Two-point Endpoint
Cal type	Spline
Sample/R1/R2	3 ul/180 ul/60 ul
Time	10 min
Sampling	16–34
Calibration	6-point calibration
Unit	U/ml
linear	80–5000

### 2.5 Neural networks and feature extraction

**FIGURE 3:**
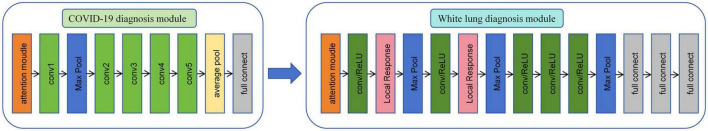
Structure of the COVID-19 White Lung Joint Diagnostic Model consists of two CNN.

As shown in Figure 3, in this study, we propose a diagnostic system composed of concatenated neural networks. To enhance the robustness and speed of the diagnostic system, we resize the chest CT images to (128 × 128 × 60). To reduce interference from other tissues on the model, we reassigned pixels greater than 600 HU to 600 and performed uniform standardization based on the range of CT attenuation values. We randomly allocate patients into training, validation, and test sets in a ratio of 3:3:4 based on negative and positive cases. We first employ a 3D-ResNet50 with an attention mechanism to classify the types of pneumonia. Then, we use a 3D-AlexNet with an attention mechanism to classify whether pneumonia will progress to white lung. In this study, we evaluate the predictive ability of the models using accuracy. Additionally, we calculate F1-score for each method. Furthermore, we perform correlation analysis between the output of the FC layer of the white lung classification network and KL-6 to provide a medical interpretation of the predictive results of the neural networks.

### 2.6 Statistics

**FIGURE 4:**
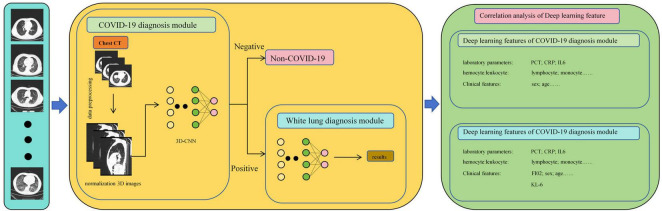
Flowchart of this study.

All analyses were performed using statistical software including SPSS (version 24.0; IBM), R (version 3.63), Python (version 3.8.5), and MedCalc (version 15.2.2). Correlation analyses were conducted using independent samples t-test or Mann-Whitney U test; Pearson or Spearman tests were used for correlation analysis between continuous categorical variables; Pearson or Fisher exact probability test was used for categorical variables. In all statistical analyses, a two-tailed *p*-value less than 0.05 was considered statistically significant. The flowchart of this study is presented in Figure 4.

## 3 Result

### 3.1 Baseline information

#### 3.1.1 Subset-I

A total of 604 patients were collected for the differential diagnosis of COVID-19 pneumonia and other pneumonias. Among them, there were 302 COVID-19 patients and 302 patients with other pneumonias. The clinical characteristics of the patients are shown in Table 2.

**TABLE 2:** Baseline Information for patients in Subset-I.

Variable*	COVID-19 patients	Non-COVID-19 patients	Statistic	*p*
Sex			2.932(X2)	0.087
M	150	171		
F	152	131		
Age(year)	56 (38, 66)	70 (63, 79)	−11.451	0
WBC(%)	6.8 (5.23, 9.68)	6.3 (4.5, 8.1)	−3.198	0.001
NEUT(%)	65.4 (56.6, 74.4)	73 (63.8, 83.7)	−6.169	0
lym(%)	23.3 (14.6, 31.1)	15.7 (8.675, 23.25)	−5.998	0
mono(%)	8 (6.6, 9.95)	7.7(5.4, 10.4)	−1.145	0.252
EO(%)	1.4 (0.425, 2.9)	0.2 (0.0, 0.925)	−9.033	0
BO(%)	0.4 (0.2, 0.6)	0.2 (0.1, 0.3)	−8.056	0
PCT(ng/mL)	0.09 (0.049, 0.158)	0.141 (0.056, 0.358)	−2.038	0.042
CRP(mg/L)	16.2 (5.82, 45.9)	33 (12.9, 77.7)	−3.532	0
IL-6(pg/mL)	18.05 (6.9, 145.1)	17.215 (8.36, 51.00)	−0.076	0.94
DD(μg/L)	190 (104, 377.5)	292 (166, 631)	−4.112	0

*BO, basophil; CRP, c-reactive protein; DD, D-Dimer; EO, eosinophil; IL-6, interleukin- 6; lmy, lymphocyte; mono, monocyte; NEUT, neutrophile; PCT, platelet; WBC, white blood cell.

Through analysis of baseline information, age and pneumonia type are correlated, indicating COVID-19 patients are present across all age groups in pneumonia cases requiring medical intervention. White blood cells, neutrophils, eosinophils, basophils, PCT, CRP, and pneumonia type are also correlated. In addition, D-dimer (DD) is correlated with pneumonia type.

#### 3.1.2 Subset-II

This dataset includes 102 COVID-19 patients, among whom 61 patients were diagnosed with white lung. The clinical characteristics of the patients are shown in Table 3.

**TABLE 3:** Baseline Information for patients in Subset-II.

Variable*	White lung patients	Non-white lung patients	Statistic	*p*
Sex			1.188(X2)	0.016
M	36	30		
F	25	31		
Age (year)	72.49 ± 10.35	67.08 ± 13.92	2.435 (t)	0.016
KL-6 (U/mL)	1100.94 ± 723	428.18 ± 385	5.891 (t)	0.002
Oxygen saturation (L/min)	3 (2, 6)	3 (2, 3)	−2.873 (Z)	0.004
FI02 (%)	33 (29, 41)	31 (27, 33)	−2.866 (Z)	0.004
PaO_2_/FiO_2_ (mmHg)	73 (62, 86)	77 (66, 115)	−1.170 (Z)	0.242
PaO_2_/FiO_2_ (most severe) (mmHg)	57.78 ± 17.33	71.96 ± 21.77	−2.987 (t)	0.517
WBC (%)	8.65 ± 7.76	6.57 ± 2.90	1.920 (t)	0.181
NEUT (%)	83 (74, 88)	70 (61, 82)	−4.233 (Z)	0
Lym (%)	8.8 (5.3, 15.1)	18.9 (9.8, 28.7)	−4.120 (Z)	0
Mono (%)	6.35 ± 2.89	8.49 ± 3.80	−3.394 (t)	0.056
EO (%)	6.0 (4.2,7.7)	0.3 (0.0, 1.2)	−3.435 (Z)	0.001
BO (%)	0.1 (0.1,0.2)	0.2 (0.1, 0.4)	−1.742 (Z)	0.081
PCT (ng/mL)	0.14 (0.08, 0.88)	0.058 (0.495, 0.415)	−0.859 (Z)	0.309
CRP (mg/L)	53.8 (37.9, 92.8)	28.8 (8.18, 84.5)	−2.364 (Z)	0.018
IL-6 (pg/mL)	38.68 ± 37.96	42.97 ± 59.84	−0.202 (t)	0.301
DD (μg/L)	358 (206, 720)	232 (134, 532)	−2.149 (Z)	0.032

*BO, basophil; CRP, c-reactive protein; DD, D-Dimer; EO, eosinophil; FI02, fraction of inspiration O2; IL-6, interleukin- 6; lmy, lymphocyte; mono, monocyte; NEUT, neutrophile; PCT, platelet; WBC, white blood cell.

Through analysis of baseline information, age correlates with the radiological manifestations of COVID-19. Oxygen uptake, oxygen concentration, and radiological manifestations of COVID-19 are related, while blood oxygen content is not correlated with radiological manifestations. This indicates that the radiological manifestations of COVID-19 correlate with the severity of respiratory failure in patients, and can be alleviated to some extent through high-concentration, high-volume oxygen therapy. D-dimer (DD), KL-6, and radiological manifestations of COVID-19 are correlated. Additionally, neutrophils, lymphocytes, eosinophils, CRP, and radiological manifestations of COVID-19 are correlated.

### 3.2 Result of deep learning

After 22 epochs, the CNN of COVID-19 diagnostic module achieved the best accuracy on the validation set. Ultimately, the network achieved an accuracy of 0.9669 and F1-score of 0.9674 on the training set. On the validation set, the network achieved an accuracy of 0.9613 and F1-score of 0.9620. On the test set, the network achieved an accuracy of 0.8884 and F1-score of 0.8911.

After 16 epochs, the CNN of white lung diagnostic module achieved the best accuracy on the validation set, which was 1.00. The network achieved an accuracy of 0.9722 and F1-score of 0.9750 on the training set. On the validation set, the network achieved an accuracy of 1.0000 and F1-score of 1.0000. On the test set, the network achieved an accuracy of 0.9184 and F1-score of 0.9286.

**FIGURE 5:**
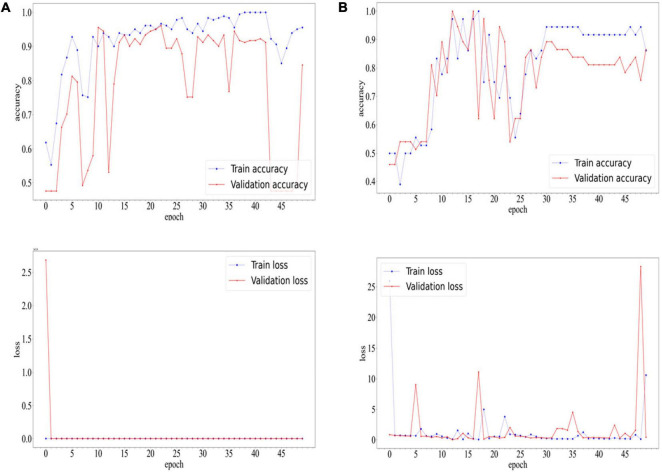
Accuracyand Loss Curves of COVID-19 diagnostic modules **(A)** and white lung diagnostic module **(B)**.

The accuracy and loss curves during the training process are shown in Figure 5.

### 3.3 Analysis of misclassification results

**FIGURE 6:**
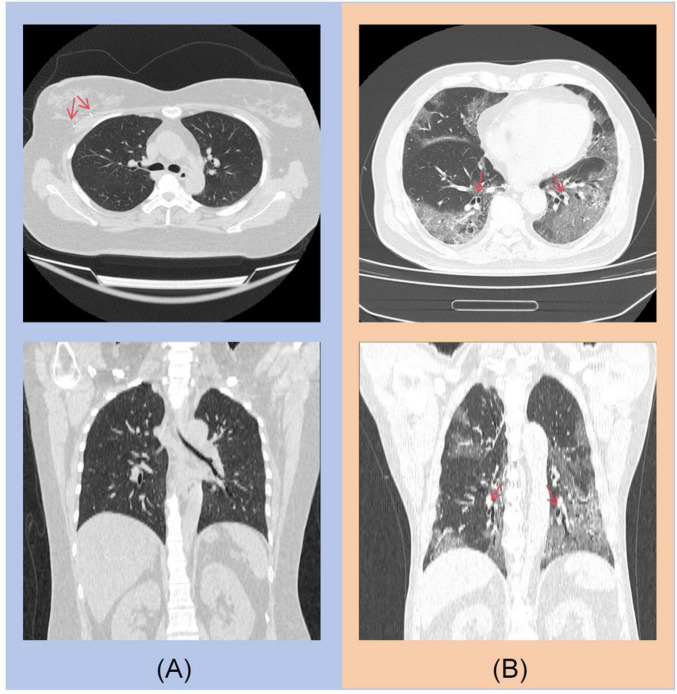
**(A)** A post-operative patient with right breast cancer, with a visible metal clip in the right breast. **(B)** An 85-year-old elderly patient with concurrent bronchiectasis.

For the COVID-19 diagnostic module, the primary errors involve misclassifying non-contrast CT scans of COVID-19 patients as other types of pneumonia. As shown in Figure 6, the main sources of misclassification are pneumonia patients with malignant tumors and elderly patients with complex lung conditions.

**FIGURE 7:**
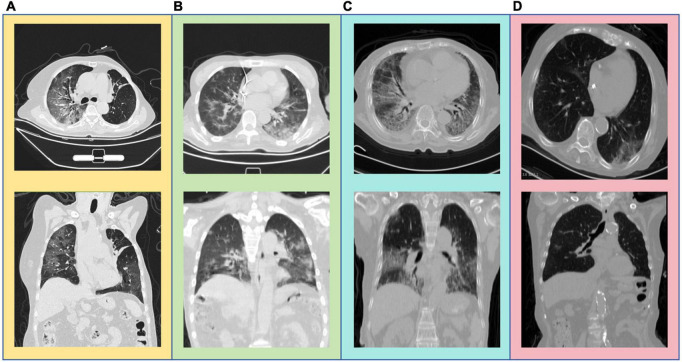
**(A)** An elderly patient with an unclear left upper lobe bronchus, distal mucus plug, and atelectasis in the lingula segment. **(B)** An elderly patient post-cardiac pacemaker surgery. **(C)** An 82-year-old elderly patient. **(D)** An 84-year-old elderly patient.

For the diagnosis module of “white lung,” the main reason for misclassifying white lung patients as ordinary COVID-19 pneumonia patients is the complexity of the lung images, or a history of chest surgery, as shown in Figures 7A, B. The primary reason for misclassifying ordinary COVID-19 pneumonia patients as white lung patients is the age of the patients, as illustrated in Figures 7C, D. These patients often have underlying lung diseases or are unable to undergo the examination in the standard supine position due to poor physical condition.

### 3.4 Correlation analysis based on laboratory parameters

In the COVID-19 diagnosis module, the correlation between deep learning features related to COVID-19 and experimental parameters is shown in Table 4. Neutrophils, eosinophils, basophils and lymphocytes were strongly correlated with the deep learning features associated with COVID-19. Compared to other types of pneumonia, deep learning features indicate that COVID-19 patients tend to have overall higher levels of white blood cells and lower proportions of neutrophils. This is consistent with the clinical characteristics of patients in Subset I and indicates that our model accurately reflects differences in white blood cells between COVID-19 and non-COVID-19 patients. Cov-related deep learning features are also associated with C-reactive protein, which is also consistent with clinical features of patients in Subset-I. Cov-related deep learning features were also associated with D-dimers, which matched clinical features of patients in Subset-I. This indicates that deep learning features can indicate whether patients have a hypercoagulable state. These results show that our deep learning features can capture information related to infection-related experimental parameters in CT images.

**TABLE 4:** Correlation Analysis of Laboratory Parameters with Deep Learning Features in the COVID-19 Diagnosis Module.

Variable*	*r*-value	*p*-value
WBC (%)	−0.078	0.090
NEUT (%)	0.304	0.000
Lym (%)	−0.294	0.000
Mono (%)	−0.062	0.177
EO (%)	−0.417	0.000
BO (%)	−0.343	0.000
PCT (ng/mL)	0.160	0.880
CRP (mg/L)	0.273	0.000
IL-6 (pg/mL)	−0.062	0.654
DD (μg/L)	−0.246	0.000

*BO, basophil; CRP, c-reactive protein; DD, D-Dimer; EO, eosinophil; IL-6, interleukin- 6; lmy, lymphocyte; mono, monocyte; NEUT, neutrophile; PCT, platelet; WBC, white blood cell.

In the white lung diagnosis module, the correlation between white lung-related deep learning features, clinical features and experimental parameters is shown in Table 5. In terms of clinical parameters, blood oxygen saturation, oxygen concentration fraction in inhaled air were associated with white lung-related deep learning features, which were consistent with clinical features of patients in Subset-II. In clinical practice, this suggests that white lung patients require a greater oxygen concentration to maintain relatively stable vital signs, and nonetheless, white lung patients also have higher levels of hypoxia than the general population. Neutrophils, eosinophils, lymphocytes, monocytes and white lung-associated deep learning features were correlated with those of patients in Subset-II. The deep learning features associated with white lung were also associated with D-dimer, and the clinical features of patients in Subset-II matched, suggesting that patients with white lung were indeed at higher risk for coagulopathy. Finally, white lung-related deep learning features were strongly associated with KL-6, also consistent with clinical features of patients in Subset-II. This suggests that patients with white lung have more severe alveolar damage than ordinary patients with COVID-19, and our model was able to capture this damage in non-enhanced CT.

**TABLE 5:** Correlation Analysis of Laboratory Parameters with Deep Learning Features in the White Lung Diagnosis Module.

Variable*	*r*-value	*p*-value
KL-6 (U/mL)	−0.422	0.000
oxygen saturation (L/min)	−0.295	0.014
FI02	−0.342	0.005
PaO2/FiO_2_ (mmHg)	0.050	0.686
PaO2/FiO_2_ (most severe) (mmHg)	0.108	0.375
WBC (%)	−0.076	0.423
NEUT (%)	−0.340	0.000
Lym (%)	0.306	0.001
Mono (%)	0.320	0.001
EO (%)	0.264	0.005
BO (%)	0.120	0.204
PCT (ng/mL)	−0.084	0.660
CRP (mg/L)	−0.233	0.030
IL-6 (pg/mL)	−0.076	0.719
DD (μg/L)	−0.293	0.020

*BO, basophil; CRP, c-reactive protein; DD, D-Dimer; EO, eosinophil; FI02, fraction of inspiration O2; IL-6, interleukin- 6; lmy, lymphocyte; mono, monocyte; NEUT, neutrophile; PCT, platelet; WBC, white blood cell.

## 4 Discussion

Lung cancer, with its high incidence and mortality rates, imposes a significant health burden on human society. The shift in the spectrum of pneumonia diseases caused by the COVID-19 pandemic undoubtedly exacerbates this burden. Compared to other pneumonias, COVID-19 spreads rapidly and poses a higher risk to lung cancer patients (28). Therefore, early diagnosis of COVID-19 through imaging provides additional value for lung cancer patients (29). In COVID-19, imaging findings precede clinical manifestations. Therefore, despite stable vital signs, severe COVID-19 diagnosed by imaging carries a high potential risk of deterioration. In the context of severe infection, patients often experience severe cardiovascular events, making resuscitation extremely challenging (30). This is especially true for lung cancer patients, whose lung function is relatively fragile (31, 32). Therefore, diagnosing COVID-19 infection and COVID-19-related severe pneumonia has significant clinical benefits for lung cancer patients.

In this study, we propose a diagnostic system consisting of two neural networks that can accurately identify COVID-19 pneumonia and other types of pneumonia. Based on this, we can predict and identify the occurrence of severe pneumonia in COVID-19 pneumonia, providing an alert for critically ill patients. Furthermore, we conducted a correlation analysis between deep learning features related to severe pneumonia and KL-6. Our predictive results show a significant correlation with KL-6, as elevated KL-6 levels are indicative of alveolar damage, demonstrating that the high-density shadows seen in imaging in COVID-19 pneumonia are directly caused by lung injury.

The identification of pneumonia types is the first step in pneumonia diagnosis and treatment. Pathogen culture is the gold standard for identifying the types of infection (33). However, this process is quite time-consuming. Therefore, for various pneumonias including COVID-19, doctors often have to rely on clinical judgment in the short term. Existing studies have been able to distinguish COVID-19 from community-acquired pneumonia, but patients who come to health care facilities often have complex infections (34). In this study, using a dataset containing 600 cases, our model achieved a accuracy rate at 0.8884, indicating its high potential for clinical application. Additionally, because our network structure is relatively straightforward, this implies that COVID-19 pneumonia exhibits significant differences compared to other types of pneumonia.

Identifying severe cases is the second step in the diagnosis and treatment of COVID-19 pneumonia. The progression of a patient’s condition and their CT imaging in COVID-19 pneumonia may not synchronize (33). For some patients, severe extensive high-density shadows on CT scans do not necessarily indicate the presence of severe respiratory failure. Due to the lack of more precise imaging biomarkers or evaluation methods, clinicians tend to subjectively interpret such CT findings as indicating a potential high risk of respiratory failure. As early as the beginning of the COVID-19 pandemic, artificial intelligence has shown tremendous potential in the diagnosis of COVID-19 (35). Based on X-rays and CT scans, researchers have used both 2D and 3D neural network models, significantly advancing the intelligence of COVID-19 diagnosis and treatment (36, 37). In this study, our neural network was able to identify patients with extensive high-density shadows with an accuracy of 0.9184, providing a potentially generalizable objective diagnostic criterion. Additionally, in this study, we found a correlation between KL-6 and the occurrence of white lung in patients, suggesting that our definition of white lung based on imaging may be caused by alveolar damage. Since alveolar damage cannot be quickly restored, this may be the reason why COVID-19 patients experience respiratory difficulties that are difficult to reverse. Compared to other types of pneumonia, COVID-19 itself carries a higher risk of hypercoagulability, and this risk is further elevated in patients with white lung. Because elderly patients are more prone to developing white lung, this increased risk may translate into sudden cardiovascular events in patients.

At the same time, our study has certain limitations: Firstly, it is a single-center study lacking external data validation. Future research needs larger sample sizes and multicenter studies to further improve the accuracy and stability of the deep learning models. Secondly, for the construction of deep learning models, there is a lack of analysis of clinical factors, which would be beneficial for building more stable models. Additionally, this study did not establish a direct link between the model and clinical outcomes, due to the data being from a specific period during the COVID-19 pandemic where patient records and treatments may not be accurate. Finally, considering the speed of diagnosis, the structure of the two diagnostic modules in this study is relatively simple, and the ability of image feature extraction is not strong. In future research, we aim to develop a universal model for classifying various pneumonias, thereby simplifying and standardizing the pneumonia diagnostic process.

## 5 Conclusion and future work

Our study results demonstrate the potential of deep learning in diagnosing COVID-19 pneumonia from CT images, particularly in distinguishing between ordinary patients and those with white lung in COVID-19 pneumonia. The 3D CNN can accomplish diagnostic tasks without manual annotation. There are differences in KL-6 expression between patients with and without white lung, while there is correlation between deep learning features associated with white lung. This suggests that patients with white lung have greater alveolar damage compared to ordinary patients. This aids in improving the prognosis of lung cancer patients with COVID-19 infection. In the future, we will develop multi-classification models for pneumonia and further explore the relevance of deep learning features to the prognosis of COVID-19 infection.

## Data availability statement

The original contributions presented in the study are included in the article/supplementary material, further inquiries can be directed to the corresponding author.

## Ethics statement

This study was approved by the Ethics Committee of Shengjing Hospital Affiliated to China Medical University (Ethics Number: 2021PS621K). Written informed consent for participation was not required for this study due to its retrospective nature, in accordance with the national legislation and the institutional requirements.

## Author contributions

TD: Conceptualization, Data curation, Formal analysis, Investigation, Methodology, Software, Validation, Visualization, Writing−original draft, Writing−review and editing. YS: Data curation, Software, Writing−review and editing. XW: Validation, Writing−review and editing. TJ: Funding acquisition, Validation, Writing−review and editing. NX: Visualization, Writing−review and editing. ZB: Funding acquisition, Methodology, Writing−review and editing. MG: Investigation, Validation, Writing−review and editing. HS: Conceptualization, Data curation, Funding acquisition, Investigation, Project administration, Supervision, Validation, Writing−review and editing. CL: Conceptualization, Data curation, Formal analysis, Funding acquisition, Investigation, Methodology, Project administration, Supervision, Validation, Writing−original draft, Writing−review and editing.
